# Bee Pollen Diet Alters the Bacterial Flora and Antimicrobial Peptides in the Oral Cavities of Mice

**DOI:** 10.3390/foods10061282

**Published:** 2021-06-04

**Authors:** Ariuntsetseg Khurelchuluun, Osamu Uehara, Durga Paudel, Tetsuro Morikawa, Yutaka Kawano, Mashu Sakata, Hiroshi Shibata, Koki Yoshida, Jun Sato, Hiroko Miura, Hiroki Nagayasu, Yoshihiro Abiko

**Affiliations:** 1 Division of Oral Medicine and Pathology, Department of Human Biology and Pathophysiology, School of Dentistry, Health Sciences University of Hokkaido, 1757 Kanazawa, Ishikari-Tobetsu, Hokkaido 061-0293, Japan; ariuntsetseg@hoku-iryo-u.ac.jp (A.K.); durga@hoku-iryo-u.ac.jp (D.P.); t-morikawa@hoku-iryo-u.ac.jp (T.M.); denty@hoku-iryo-u.ac.jp (K.Y.); j-sato@hoku-iryo-u.ac.jp (J.S.); 2 Division of Oral and Maxillofacial Surgery, Department of Human Biology and Pathophysiology, School of Dentistry, Health Sciences University of Hokkaido, 1757 Kanazawa, Ishikari-Tobetsu, Hokkaido 061-0293, Japan; nagayasu@hoku-iryo-u.ac.jp; 3 Division of Disease Control and Molecular Epidemiology, Department of Oral Growth and Development, School of Dentistry, Health Sciences University of Hokkaido, 1757 Kanazawa, Ishikari-Tobetsu, Hokkaido 061-0293, Japan; osamu@hoku-iryo-u.ac.jp (O.U.); hmiura@hoku-iryo-u.ac.jp (H.M.); 4 Institute of Preventive Medical Science, Health Sciences University of Hokkaido, Ainosato 2-5, Kita-ku, Sapporo, Hokkaido 002-8072, Japan; ykawano@hoku-iryo-u.ac.jp; 5 Belle Coeur Institute, Utsukushigaoka 5-9-10-30, Kiyota-ku, Sapporo, Hokkaido 004-0851, Japan; m.sakata@belle-coeur.jp (M.S.); h.shibata@belle-coeur.jp (H.S.)

**Keywords:** antimicrobial peptides, bee pollen, metagenomics, oral cavity

## Abstract

Background: Bee pollen (BP) has a broad range of beneficial effects on health. The aim of this study was to examine the effect of BP on the oral environment, including the microbiome and antimicrobial peptides. Methods: C57BL/6J mice were randomly divided into two groups: control and BP. The BP group was fed with a 5% BP diet for 1 month. Swabs from the oral and buccal mucosa and samples of the intestinal stool were collected. Genomic DNA was extracted and the microbiome was analyzed via 16S rRNA sequencing. Results: BP inhibited the growth of *P. gingivalis* at a concentration of >2.5%. The metagenomic study showed that the abundance of genus *Lactococcus* was significantly elevated in the oral and intestinal microbiomes of the BP group when compared to those of the control group. Significant alterations in alpha and beta diversity were observed between the oral microbiomes of the two groups. The mRNA levels of beta-defensin-2 and -3 were significantly upregulated in the buccal mucosa of the BP group. Conclusion: A BP diet may have a beneficial effect on oral and systemic health by modulating the bacterial flora and antimicrobial peptides of the oral cavity. Further investigations are needed to clarify how a BP diet affects overall human health.

## 1. Introduction

Bee pollen (BP) is a pollen ball or pellet that is carried by the honey bee while collecting honey. The chemical components of BP depend on the type of flowers the pollen is collected from. However, it is known to contain protein (5–60%), reducing and non-reducing sugars (13–55%), lipids (4–7%), crude fiber (0.3–20%), minerals and bioactive substances (including vitamins), enzymes, and phenolic compounds [1]. The protein contents include essential amino acids such as methionine, lysine, threonine, histidine, leucine, isoleucine, valine, phenylalanine, and tryptophan [2,3]; the lipids include essential fatty acids such as linoleic, **γ**-linoleic and archaic acids, and P-sitosterol [2], and the phenolic compounds include flavonoids (kaempferol, quercetin, and isorhamnetin), leukotrienes, catechins, and phenolic acid [2].

BP has a broad range of beneficial effects, including antioxidant, antifungal, antimicrobial, antiviral, anti-inflammatory, immuno-stimulating, antitumor, and local analgesic effects on health [1,2]. Many studies have demonstrated the antioxidant activity of BP, which contributes to the prevention of certain illnesses and the protection of cells and tissues against damage by oxidative stresses [4]. Based on these reports, several BP products are commercially available in the form of supplements, ingredients, candies, and cosmetics. Furthermore, many of these products can be orally administered, often repeatedly. Nonetheless, the effect of BP on the oral environment has not been demonstrated so far. The oral cavity is lined by the oral mucosa and consists of saliva, which harbors more than 770 species of bacteria [5,6]. Most of these species form a symbiotic biofilm that is important for defending against pathogenic bacteria, controlling inflammation, and maintaining homeostasis [7]. Recently, evidence has been growing of the involvement of oral bacteria in systemic diseases, such as pneumonia, cardiovascular diseases, diabetes, and autoimmune diseases [8]. In addition, oral microbes can be ingested and will naturally translocate to the digestive tract, where they can potentially form ectopic colonies in the upper and lower digestive tracts; this ectopic colonization has been suggested to induce the development of digestive diseases [5]. The oral administration of BP might affect human health via alterations in oral and intestinal microbes, and the direct effect of BP on the oral mucosa and both oral and intestinal microbes should therefore be investigated. The antimicrobial activities of BP and the epithelial antimicrobial peptides produced by the oral mucosa might affect the oral microbe. Bioactive natural components such as epigallocatechin-3-gallate in green tea have been shown to upregulate the expression levels of epithelial antimicrobial peptides [9,10].

The aim of the present study is to examine the effect of BP on the oral environment, including the bacterial flora, and on the expression of antimicrobial peptides in the oral mucosa. The antimicrobial activities against oral bacteria, including *Streptococcus mutans* and *Porphyromonas gingivalis*, were assessed in vitro. Furthermore, alterations in oral and intestinal flora were analyzed by next-generation sequencing, and the expression levels of epithelial antimicrobial peptides, including beta-defensins, cathelicidin, and calprotectin, were examined in vivo.

## 2. Materials and Methods

**Figure 1:**
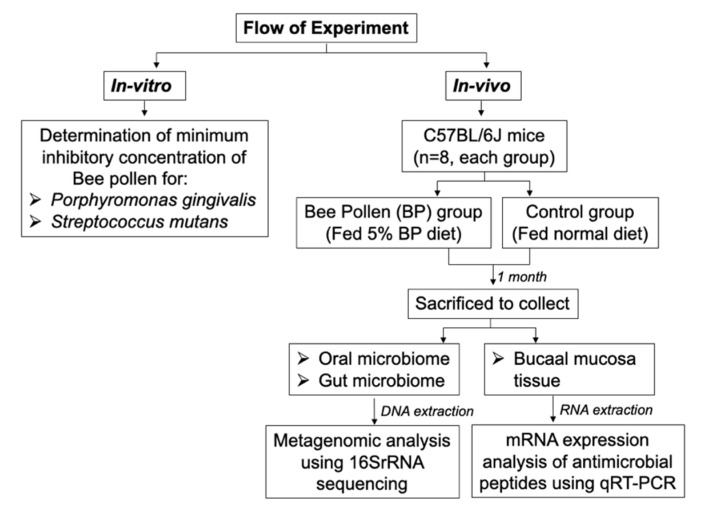
Schematic flow of the experiment.

The schematic flow of the experiment is shown in Figure 1.

The minimum inhibitory concentration (MIC) of BP on two oral bacteria, *Streptococcus mutans* (ingbritt strain) and *Porphyromonas gingivalis* (ATCC33277 strain), was examined. Hemin and menadione were added to Brain Heart Infusion (BHI; Becton, Dickinson and Company, Franklin Lakes NJ, USA) liquid medium and incubated at 37 °C under anaerobic conditions (80% N_2_, 10% H_2_, and 10% CO_2_). *S. mutans* and *P. gingivalis* were pre-cultured in BHI liquid medium; cells were collected, washed, and the absorbance (OD) of the bacterial solution was measured with a spectrophotometer at a wavelength of 600nm. The concentration of the bacterial solution was adjusted to OD = 1. The BP extract (API Co., Ltd., Gifu, Japan) was diluted with BHI medium. The dilution series of BP was prepared in a 96-well plate, and the bacterial solution was inoculated at a dilution of 1:100 in BHI liquid medium. The MIC was determined after 72 h of anaerobic culture.

### 2.1. Animals

All procedures involving animals were performed according to the Regulations for the Care and Use of Laboratory Animals at the Health Sciences University of Hokkaido. This experimental protocol was approved by the animal experimental and ethics committee of the Health Sciences University of Hokkaido (Permission number: 20-053). BP was obtained from API Co., Ltd. (Gifu, Japan) and a diet was prepared by mixing 5% BP with the standard laboratory chow (Oriental Yeast, Tokyo, Japan). Six-week-old (male) C57BL/6J mice were randomly divided into a control and a BP group (*n* = 8 in each group). The mice in the BP group were fed with the BP diet for 1 month, while the control group received the standard laboratory chow. After a month, both groups were anesthetized by intraperitoneal injection of sodium pentobarbital (50 mg/kg). The oral microbiome was collected using a swab; the oral cavity was swabbed for 30 s, starting from the dorsum of the tongue to the palate, followed by the buccal mucosa, the upper and lower vestibules, and the floor of the mouth. The swab was then placed in 1.5 mL tubes containing 200 µL Tris-EDTA buffer (10 mM Tris-HCl, 0.1 mM EDTA, pH: 8.0) and frozen at −80 °C until further analysis. The intestinal stool was collected from the terminal part of the large intestine and immediately frozen at −80 °C until further processing.

### 2.2. Bacteria Collection and DNA Extraction

Genomic DNA was extracted from the oral swab and samples using the DNeasy Blood & Tissue Kit (Qiagen, Hilden, Germany) and DNeasy PowerSoil Kit (Qiagen), respectively, according to the manufacturer’s instructions. DNA extracts were stored at −20 °C and used for metagenomic analysis.

### 2.3. Sequencing and Library Preparation

The amplicon polymerase chain reaction (PCR) targeted the V3–V4 regions of the bacterial 16S ribosomal RNA (rRNA) gene. Sequencing libraries of the V3–V4 region were generated according to the 16S Metagenomic Sequencing Library Preparation protocol (Illumina, San Diego, CA, USA). In brief, the V3–V4 regions of the 16S bacterial rRNA gene were amplified using a two-step PCR protocol. The KAPA HiFi HS ReadyMix (Nippon Genetics, Tokyo, Japan) and V3–V4 region primers were used for the amplicon PCR, and the KAPA HiFi HS ReadyMix and Nextera XT index kit (Illumina) were used for the index PCR. The libraries were purified using AM Pure XP (Beckman Coulter, MA, USA) and quantified using a Qubit 3 fluorometer (Thermo Fisher Scientific, Waltham, MA, USA). Subsequently, the library was diluted, mixed with PhiX (Illumina), and subjected to sequencing using a MiSeq reagent kit v3 (600 cycles, Illumina) and a MiSeq system (Illumina).

### 2.4. Analysis of Sequencing Data

The metagenomic sequencing data were analyzed using the software package Quantitative Insights into Microbial Ecology2 (QIIME2, v2020.2) against the 16S rRNA gene sequences that were assigned to the 16S rDNA database (Greengenes v13.8). The alpha diversity, based on identified operational taxonomic units (OTUs), was estimated using the observed OTUs and the Shannon-group significance. To account for multiple comparisons at each taxonomic level, a Benjamini–Hochberg false discovery rate (FDR)-adjusted *p*-value (q-value) was used. The beta diversity was evaluated based on the UniFrac distance, which represented the fraction of the branch length of the phylogenetic tree that is shared between groups. Three-dimensional principal coordinate analysis (PCoA) was used to generate UniFrac scatterplots to visually compare the microbial composition across groups. Differences in bacterial communities between the control and BP groups were analyzed using the weighted and unweighted UniFrac distance metric. Permutational multivariate analysis of variance (PERMANOVA) was used for the weighted and unweighted UniFrac distance matrices to determine significant differences in microbial communities between the two groups. A *p*-value of <0.05 was considered statistically significant. Significant differences in microbial taxa abundance between the control and BP groups were analyzed using the analysis of comparison of microbiome (ANCOM) test in the QIIME2. The final significance is expressed in the empirical distribution of W.

### 2.5. Tissue Collection and RNA Extraction and Quantitative Reverse Transcriptase-PCR

Total RNA was extracted from the oral tissues (buccal mucosa) using TRIzol Reagent (Thermo Fisher Scientific, MA, USA), according to the manufacturer’s instructions. The RNA extracts were stored at −80 °C and used for mRNA expression analysis. The extracted RNA was reverse transcribed to cDNA using ReverTra Ace^®^ qPCR RT Master Mix (Toyobo, Osaka, Japan). The mRNA expression levels were measured using LightCycler^®^ 96 (Roche Diagnostics, Basel, Switzerland). Table 1 illustrates the primer sequences used in this study. Quantitative reverse transcriptase-PCR (qRT-PCR) was performed in reactions containing the obtained cDNA, KAPA SYBR FAST qPCR Mix (Nippon Genetics, Tokyo, Japan), and a pair of each primer. The PCR conditions included the following steps: initial pre-incubation at 95 °C for 3 min, denaturation at 95 °C, 40 cycles of denaturation at 95 °C for 10 s, annealing at 60 °C for 20 s, and elongation at 72 °C for 1 s. The relative expression levels of each mRNA were calculated as the Cq (value obtained by subtracting the Cq value of GAPDH mRNA from the Cq value of the target mRNA) using the ∆∆Cq method. Specifically, the amount of target mRNA relative to the GAPDH mRNA was expressed as 2-(∆Cq). Data are expressed as the ratio of the target mRNA to the GAPDH mRNA. Statistical analysis of the gene expression levels was performed using SPSS version 26 software (SPSS Inc., Chicago, IL, USA). The results were compared using the Mann–Whitney U test. Data are presented as mean ± standard error, and a *p* < 0.05 was considered significant (*n* = 8).

## 3. Results

### 3.1. Minimum Inhibitory Concentration

**Figure 2:**
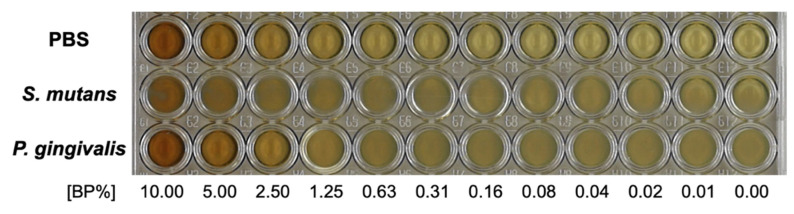
The minimum inhibitory concentrations (MIC) of the bacteria. *P. gingivalis* showed growth inhibition at a bee pollen concentration ranging from 2.5 to 10%.

*S. mutans* showed no growth inhibition when the BP concentrations ranged from 0 to 10.0%. No *P. gingivalis* growth was noticed at concentrations ranging from 2.5 to 10.0%; therefore, 2.5% was considered as the MIC of BP for *P. gingivalis* (Figure 2).

### 3.2. Oral and Gut Bacterial Species Richness and Diversity (Alpha Diversity)

**Figure 3:**
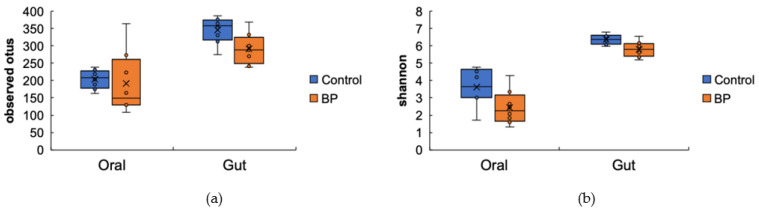
Richness and diversity of the oral and gut bacterial species (alpha diversity). (**a**) The observed OTUs of gut bacterial flora were significantly higher in the control group than in the BP group (*p* = 0.027; *q* = 0.027). (**b**) The Shannon of oral and gut bacterial flora was significantly higher in the control group than in the BP group (*p* = 0.045; *q* = 0.045).

The alpha diversity was ascertained to evaluate the different types of oral and gut bacterial flora present in each group. No significant differences in the observed OTUs of the oral bacterial flora were observed between the control and BP groups (*p* = 0.345; *q* = 0.345; Figure 3a). The Shannon of the oral bacterial flora was significantly higher in the control group than in the BP group (*p* = 0.045, *q* = 0.045; Figure 3b). The observed OTUs (*p* = 0.027; *q* = 0.027; Figure 3a) and the Shannon (*p* = 0.045; *q* = 0.045; Figure 3b) of the gut bacterial flora were significantly higher in the control group than in the BP group.

### 3.3. Oral and Gut Bacterial PCoA of the Weighted and Unweighted UniFrac (Beta Diversity)

**Figure 4:**
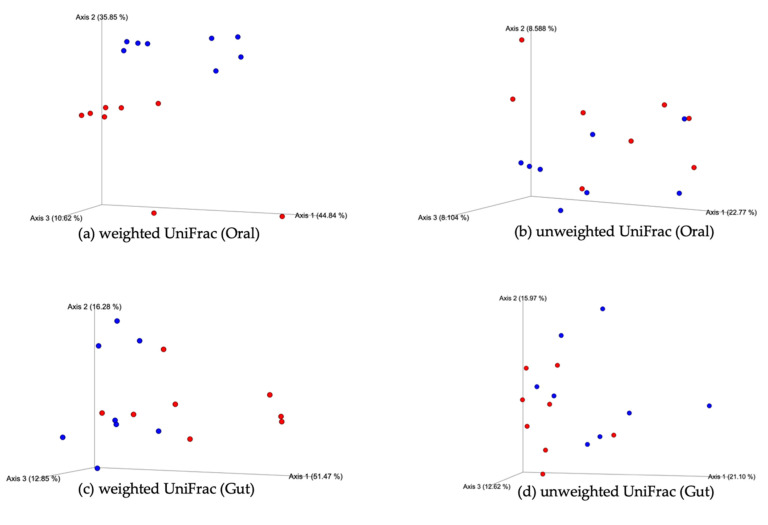
PCoA of the weighted and unweighted UniFrac (beta diversity) distances in the oral and gut bacteria. (**a**,**b**) The weighted UniFrac distances of the oral microbiome significantly differed between the control (blue dots) and BP groups (red dots) (PERMANOVA; *p* = 0.001; *q* = 0.001). (**c**,**d**) The weighted (*p* = 0.006; *q* = 0.006) and unweighted UniFrac (*p* = 0.032; *q* = 0.032) distances of the gut microbiome significantly differed between the control and BP groups.

The PCoA of the UniFrac distance was analyzed to evaluate the difference in the diversity of the oral flora between the two groups. The PCoA plots demonstrated clustering between the oral flora of the control and BP groups. The weighted UniFrac distances were significantly different between the two groups (*p* = 0.001; *q* = 0.001; Figure 4a). In contrast, no significant differences were observed in the unweighted UniFrac distances between the two groups (*p* = 0.295; *q* = 0.295; Figure 4b). Similarly, the PCoA plots demonstrated clustering between the gut flora of the control and BP groups. The weighted (*p* = 0.006; *q* = 0.006) and unweighted (*p* = 0.032; *q* = 0.032; Figure 4c,d) UniFrac distances significantly differed between the two groups.

### 3.4. Oral and Gut Bacterial Taxonomy

**Figure 5:**
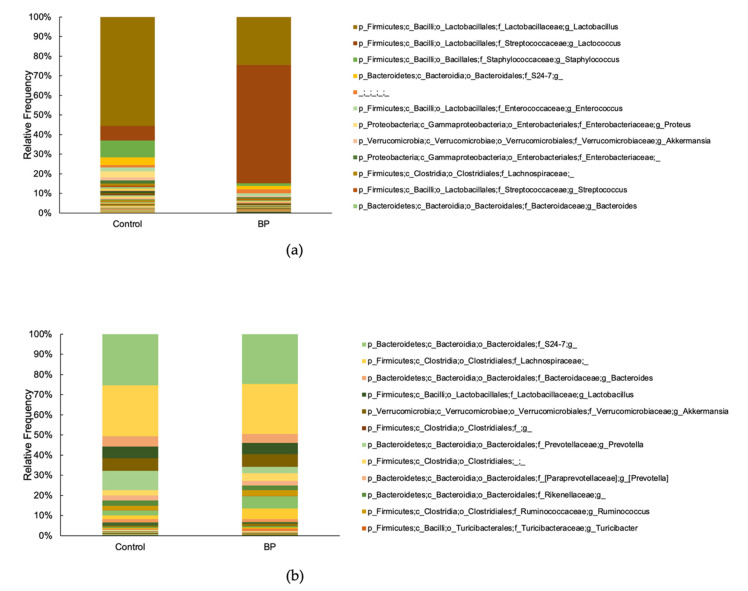
Oral and gut bacterial taxonomy. (**a**) The most abundant genus in the oral microbiome was Lactobacillus, followed by Lactococcus and Staphylococcus. (**b**) The most abundant genus in the gut microbiome was *S24-7;g*, followed by Lachnospiraceae and Bacteroides.

Sixteen samples were sequenced using MiSeq, and a total of 1,291,228 sequences were amplified from the oral flora of the control and BP groups (range, 28,436–114,903 sequences per sample; mean, 80,701 sequences per sample). A total of 221 different bacterial genera were detected in the two groups using QIIME2. The most abundant genus among all the samples was *Lactobacillus*, followed by *Lactococcus* and *Staphylococcus* (Figure 5a). The ANCOM test revealed one differentiating genus between the oral flora of the control and BP groups: *Lactococcus* (*W* = 82). This genus had a higher proportion in the BP group than in the control group (Table 2).

Likewise, 16 samples were sequenced, and a total of 1,481,501 sequences were amplified from the gut flora of the control and BP groups (range, 56,551–118,116 sequences per sample; mean, 92,593 sequences per sample). A total of 102 different bacterial genera were detected in the gut flora from the two groups. The most abundant genus among all samples was *S24-7;g*, followed by *Lachnospiraceae* and *Bacteroides* (Figure 5b). At the genus level, the ANCOM test revealed 102 differentiating genera between the control and BP groups, and the most increased bacteria at the genus level was *Turicibacter* (*W* = 18; Table 2).

### 3.5. Gene Expression Levels in the Oral and Gut Tissues

**Figure 6:**
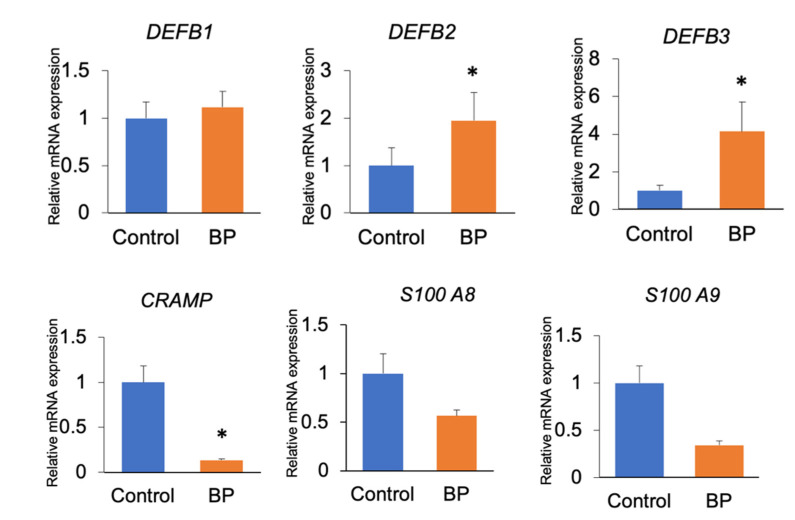
Relative mRNA expression levels of AMPs. DEFB2 and DEFB3 were significantly upregulated, whereas CRAMP (cathelicidin) was significantly downregulated in the buccal mucosae of mice from the BP group when compared to those in the control group (* *p* < 0.05). Data are expressed as mean and the error bars represent the standard error.

The mRNA expression levels of beta-defensin-2 (DEFB2) and beta-defensin-3 (DEFB3) were significantly upregulated, whereas that of cathelicidin-related antimicrobial peptide (CRAMP) was significantly downregulated in the buccal mucosae of mice belonging to the BP group when compared to those in the control group (*p* < 0.05). The mRNA expression levels of other antimicrobial peptides, such as beta-defensin-1 (DEFB1), S100 A8, and S100 A9, showed no significant differences between the two groups (Figure 6).

## 4. Discussion

The present study demonstrates the differences in the abundance and diversity of the oral flora between the BP and control groups using 16S rRNA sequencing. The proportion of the *Lactococcus* genus dramatically increased in the oral microbe of the BP group. *Lactococcus lactis* has an inhibitory effect on *S. mutans* [11]; in addition, it prevents the formation of biofilms that contain *P. gingivalis* [12]. As such, BP might have a beneficial effect on oral health by increasing the proportion of *Lactococcus*. The significantly higher proportion of *Lactococcus* in the gut microbe of mice in the BP group was confirmed by the ANCOM analysis. Previous human and animal studies have shown that certain strains of *Lactococcus* can survive within the gut and reach the gastrointestinal tracts [13,14]. *Lactococcus* is a probiotic with several beneficial effects on human health, such as a reduction in serum cholesterol levels, an improvement in the balance of the intestinal microflora, immunomodulatory activities, and improvements in skin health [15]. The increased proportion of *Lactococcus* in the oral microflora might have a beneficial effect on both systemic and oral health.

We speculated that the alterations in the proportions of genera in the oral flora were due to the antimicrobial activities of BP alone or in conjunction with the antimicrobial peptides that were produced due to the BP. The antimicrobial activities of BP against certain types of bacteria have been reported previously [16]; therefore, this study aimed to examine the antimicrobial activities of BP against *S. mutans*, *P. gingivalis*, and other oral pathogens in vitro. Although no antimicrobial activities were observed against *S. mutans*, BP was found to inhibit the growth of *P. gingivalis*. *S. mutans* plays a role in tooth decay by metabolizing sucrose; on the other hand, *P. gingivalis* is non-asaccharolytic and a potent proteolytic bacterium [17]. BP contains various amounts of reducing and non-reducing sugars [1], which might aid in the growth of *S. mutans*. Furthermore, BP can exert an antimicrobial effect on *P. gingivalis* in the absence of any saccharolytic activities. This antimicrobial activity of BP might be involved in the alteration of the proportion of genera in the oral flora.

BP was found to affect the expression levels of antimicrobial peptides—including beta-defensins, calprotectins (S100A8 and S100A9), and cathelicidin—produced by the oral epithelium. BP upregulated the expression levels of *DEFB2* and *DEFB3* and downregulated the expression of *CRAMP* in the oral mucosal tissues. The expression dynamics of these antimicrobial peptides might vary, despite being produced within the oral epithelium. Beta-defensin-1 is constitutively expressed, whereas beta-defensin-2 and -3 are inducible by stimulation with inflammatory cytokines and certain types of bacteria [18]. The expression of calprotectin is induced by stimulation with IL-1alpha, but not by lipopolysaccharides. Although cathelicidin/LL-37 can be found in the gingival epithelium, its expression appears to be a product of neutrophil migration in epithelial cells, rather than production [19]. The downregulated expression of cathelicidin in the present study may be due to the lower numbers or decreased functions of the neutrophils. Antimicrobial peptides have broad-spectrum antimicrobial activities against Gram-positive and Gram-negative oral bacteria and *Candida* [20]. Previously, human beta-defensin-2 has exhibited potent antimicrobial activity against Gram-negative bacteria and *Candida*, but not *Staphylococcus aureus* [21]. The antimicrobial activities of human beta-defensin-2 and -3 might vary among bacterial species. Two mechanisms are thought to be involved in the upregulated expression levels of beta-defensin-2 and -3 in the present study: the effectiveness of BP on the oral mucosa, and the alterations in the proportions of the genera due to the BP. The effects of BP on other types of cells, including white blood cells, have been shown previously [16]. BP compounds such as polyphenols or flavonoids might have beneficial effects on white blood cells. The anti-inflammatory action of flavonoids might be attributed to the activity of quercetin, which is known to decrease the level of arachidonic acid leading to reduced levels of proinflammatory prostaglandins [16]. The neutrophils in saliva are mainly derived from the gingival sulcus; they migrate through the junctional epithelium and out of the gingival sulcus in healthy gingiva. Inflammatory regulators, including IL-8 and an intercellular adhesion molecule-1 receptor, play a role in neutrophil migration [22]. Thus, the anti-inflammatory effect of BP on neutrophils might result in a decrease in the number of neutrophils and lead to a downregulation in the expression level of cathelicidin. Furthermore, BP is recognized as a potent inhibitor of tumor necrosis factor (TNF)-induced nuclear factor (NF)-κB activation [23]. TNF-induced NF-κB activation upregulates the expression levels of human beta-defensin-2 and -3 [24]. Hence, the anti-inflammatory action of BP via NF-κB activation might suppress this upregulated expression.

The limitation of this study is that we could not demonstrate whether the altered expression in AMPs was directly induced by the BP, or as a result of alterations in the proportions of bacteria present due to the BP. The proportion of *Lactococcus* was highest in the BP group. Recently, *Lactococcus* was reported to improve the functions of the epithelial barrier and increase the expression level of beta-defensin-2 in human skin [25], which may support our speculation that increased proportions of *Lactococcus* by BP induce the upregulation of both beta-defensin-2 and -3. Moreover, in our study, we could not demonstrate whether the BP directly caused the alteration of microbiomes, or whether this occurred via altered AMPs. The alterations in the proportions of the genera in the oral flora might be due to the antimicrobial activities of BP alone, or may be in conjunction with the epithelial antimicrobial peptides that are produced as a result of the alteration of genera by BP. Further investigations are required to determine how BP increases the proportion of *Lactococcus*.

## 5. Conclusions

This study demonstrates that BP increased the proportion of *Lactococcus* in the oral cavities of mice, alongside an increase in the expression levels of beta defensin-2 and -3. *Lactococcus*, and the beta-defensins play inhibitory roles on oral pathogens. Furthermore, *Lactococcus* might translocate to the digestive tract and function as a probiotic. These findings indicate that BP might have beneficial effects on both oral and systemic health.

## Figures and Tables

**Table 1:** The primer sequences used in this study (5′–3′).

*DEFB1*	F: CCAGATGGAGCCAGGTGTTG
	R: AGCTGGAGCGGAGACAGAATCC
*DEFB2*	F: AAGTATTGGATACGAAGCAG
	R: TGGCAGAAGGAGGACAAATG
*DEFB3*	F: GCATTGGCAACACTCGTCAGA
	R: CGGGATCTTGGTCTTCTCTA
*CRAMP*	F: GGCGGTCACTATCACTGCTG
	R: TCGGAACCTCACAGACTTGG
*S100A8*	F: TGCCCTCTACAAGAATGACT
	R: AAGCTCTGCTACTCCTTGTG
*S100A9*	F: CGACACCTTCCATCAATACT
	R: TCAGCATCATACACTCCTCA
*GAPDH*	F: AGAACATCATCCCTGCATCC
	R: CACATTGGGGGTAGGAACAC

**Table 2:** The ANCOM results and percentile abundances of features in each group.

	Median Percentile Abundance		Max Percentile Abundance		
	Control	BP	Control	BP	W
**Oral flora of genus** p_Firmicutes;c_Bacilli;o_Lactobacillales;f_Streptococcaceae;g_Lactococcus	2205.5	56,198	22,665	86,182	82
**Gut flora of genus** p_Firmicutes;c_Bacilli;o_Turicibacterales;f_Turicibacteraceae;g_Turicibacter	3671	582	8853	2999	18
p_Firmicutes;c_Erysipelotrichi;o_Erysipelotrichales;f_Erysipelotrichaceae;g_Coprobacillus	7.5	76	48	414	4
p_Firmicutes;c_Bacilli;o_Lactobacillales;f_Streptococcaceae;g_Lactococcus	1	3	1	10	4
p_Firmicutes;c_Clostridia;o_Clostridiales;f_Lachnospiraceae;g_Clostridium	12.5	1	34	5	3
_;_;_;_;_	240	69.5	678	251	2
p_Bacteroidetes;c_Flavobacteriia;o_Flavobacteriales;f_Flavobacteriaceae;g_Capnocytophaga	1	1	1	3	2
p_Firmicutes;c_Clostridia;o_Clostridiales;f_Veillonellaceae;g_Selenomonas	1	1	1	6	2
p_Proteobacteria;c_Gammaproteobacteria;o_Pseudomonadales;f_Moraxellaceae;g_Acinetobacter	1	1	1	19	2
p_TM7;c_TM7-3;o_;f_;g_	1	1	1	7	2
p_Firmicutes;c_Erysipelotrichi;o_Erysipelotrichales;f_Erysipelotrichaceae;g_Clostridium	1	1	1	3	1
p_Proteobacteria;c_Gammaproteobacteria;o_Pasteurellales;f_Pasteurellaceae;g_Aggregatibacter	1	1	3	5	1
p_Firmicutes;c_Clostridia;o_Clostridiales;f_Ruminococcaceae;g_	743	1164.5	1644	2639	1
p_Firmicutes;c_Clostridia;o_Clostridiales;f_Dehalobacteriaceae;g_Dehalobacterium	177	100	328	449	1
p_Firmicutes;c_Clostridia;o_Clostridiales;f_Christensenellaceae;g_	1	1	3	1	1
p_Tenericutes;c_Mollicutes;o_RF39;f_;g_	32.5	64.5	163	129	1
p_Firmicutes;c_Bacilli;o_Lactobacillales;f_Lactobacillaceae;g_Lactobacillus	8426.5	2474	15,887	8004	1
p_Deferribacteres;c_Deferribacteres;o_Deferribacterales;f_Deferribacteraceae;g_Mucispirillum	120.5	23.5	2403	55	1
p_Firmicutes;c_Clostridia;o_Clostridiales;f_Peptostreptococcaceae;g_Peptostreptococcus	1	1	19	13	1
p_Bacteroidetes;_;_;_;_	1	1	3	1	1
p_Bacteroidetes;c_Bacteroidia;o_Bacteroidales;_;_	1	1	1	3	1
p_Bacteroidetes;c_Bacteroidia;o_Bacteroidales;f_Bacteroidaceae;g_Bacteroides	7508.5	5739.5	9575	7975	1
p_Bacteroidetes;c_Bacteroidia;o_Bacteroidales;f_Rikenellaceae;_	79	59.5	119	139	1
p_Bacteroidetes;c_Bacteroidia;o_Bacteroidales;f_Rikenellaceae;g_	4200	1955	5724	5190	1
p_Bacteroidetes;c_Bacteroidia;o_Bacteroidales;f_S24-7;g_	31,801.5	25,334.5	39,150	31,959	1
p_Bacteroidetes;c_Bacteroidia;o_Bacteroidales;f_Porphyromonadaceae;g_Parabacteroides	921.5	645	1406	976	1
p_Bacteroidetes;c_Bacteroidia;o_Bacteroidales;f_[Paraprevotellaceae];g_[Prevotella]	3224	3911.5	3355	7706	1

## Data Availability

The data is available on reasonable request to the corresponding author.
